# Diabetes and Prostate Cancer Outcomes in Obese and Nonobese Men After Radical Prostatectomy

**DOI:** 10.1093/jncics/pkab023

**Published:** 2021-03-09

**Authors:** Sonia Kelkar, Taofik Oyekunle, Adva Eisenberg, Lauren Howard, William J Aronson, Christopher J Kane, Christopher L Amling, Matthew R Cooperberg, Zachary Klaassen, Martha K Terris, Stephen J Freedland, Ilona Csizmadi

**Affiliations:** 1 Urology Section, Veterans Affairs Medical Center, Durham, NC, USA; 2 Duke Cancer Institute Biostatistics Shared Resource, Duke University School of Medicine, Durham, NC, USA; 3 Department of Medicine, Division of Endocrinology, Duke University Medical Center, Durham, NC, USA; 4 Department of Urology, University of California Los Angeles Medical Center, Los Angeles, CA, USA; 5 Urology Section, Wadsworth VA Medical Center, Los Angeles, CA, USA; 6 Department of Urology, University of California San Diego Health System, San Diego, CA, USA; 7 Department of Urology, Oregon Health & Science University, Portland, OR, USA; 8 Department of Urology, University of California San Francisco Medical Center, San Francisco, CA, USA; 9 Department of Surgery, Section of Urology, Augusta University, Augusta, GA, USA; 10 Department of Surgery, Division of Urology, Cedars-Sinai Medical Center, Los Angeles, CA, USA

## Abstract

**Background:**

The link between diabetes and prostate cancer progression is poorly understood and complicated by obesity. We investigated associations between diabetes and prostate cancer–specific mortality (PCSM), castrate-resistant prostate cancer (CRPC), and metastases in obese and nonobese men undergoing radical prostatectomy (RP).

**Methods:**

We included 4688 men from the Shared Equal Access Regional Cancer Hospital cohort of men undergoing RP from 1988 to 2017. Diabetes prior to RP, anthropometric, and clinical data were abstracted from 6 Veterans Affairs Medical Centers electronic medical records. Primary and secondary outcomes were PCSM and metastases and CRPC, respectively. Multivariable-adjusted hazard ratios (adj-HRs) and 95% confidence intervals (CIs) were estimated for diabetes and PCSM, CRPC, and metastases. Adjusted hazard ratios were also estimated in analyses stratified by obesity (body mass index: nonobese <30 kg/m^2^; obese ≥30 kg/m^2^). All statistical tests were 2-sided.

**Results:**

Diabetes was not associated with PCSM (adj-HR = 1.38, 95% CI = 0.86 to 2.24), CRPC (adj-HR = 1.05, 95% CI = 0.67 to 1.64), or metastases (adj-HR = 1.01, 95% CI = 0.70 to 1.46), among all men. Interaction terms for diabetes and obesity were statistically significant in multivariable models for PCSM, CRPC, and metastases (*P* ≤ .04). In stratified analyses, in obese men, diabetes was associated with PCSM (adj-HR = 3.06, 95% CI = 1.40 to 6.69), CRPC (adj-HR = 2.14, 95% CI = 1.11 to 4.15), and metastases (adj-HR = 1.57, 95% CI = 0.88 to 2.78), though not statistically significant for metastases. In nonobese men, inverse associations were suggested for diabetes and prostate cancer outcomes without reaching statistical significance.

**Conclusions:**

Diabetes was associated with increased risks of prostate cancer progression and mortality among obese men but not among nonobese men, highlighting the importance of aggressively curtailing the increasing prevalence of obesity in prostate cancer survivors.

Decades of epidemiologic and clinical research reveal a complex relation between diabetes and prostate cancer (1). Whereas diabetes is associated with an increased risk in most cancers (eg, liver, pancreas, breast, colorectal, endometrial, bladder, non-Hodgkin lymphoma) (2), it has largely been reported to be protective for prostate cancer (3-6); however, null results have also been reported (7‐9). Studies suggest that lower levels of prostate-specific antigen (PSA), characteristic of diabetes, and/or antidiabetic medications may mask prostate cancer, leading to an underdiagnosis and protective association (1,10). Additionally, a decrease in prostate biopsy referrals has recently been reported to contribute to the appearance of a protective effect in men taking antidiabetic medications (11).

Most studies have focused on prostate cancer incidence (6,12‐14), with fewer studies examining the relation between diabetes and prostate cancer progression (15,16). Moreover, the modifying roleof obesity has not been well studied. Growing concerns about obesity are especially relevant in the context of prostate cancer, specifically aggressive prostate cancer and high-grade tumors, because of its increasing prevalence and association with diabetes and because there are few modifiable risk factors for prostate cancer (17‐19). Furthermore, obesity itself is associated with an increased risk of prostate cancer progression (20‐23).

We previously reported that in men undergoing radical prostatectomy (RP), diabetes was associated with an increased risk of biochemical recurrence (24) and metastases (15) in obese men, but not in nonobese men. Furthermore, we reported that in diabetic men, longer duration of diabetes was associated with an increased risk of metastases (15). Hence, we hypothesized that diabetes at RP would be associated with an increased risk of prostate cancer–specific mortality (PCSM) in obese men, but not in nonobese men. Because our earlier studies were limited in sample size and follow-up, we now repeat the analysis with a larger cohort and longer follow-up.

The primary objectives of this study were to investigate the association between diabetes and PCSM in men undergoing RP and to examine if the association was modified by obesity. In secondary objectives, we studied associations between diabetes and risks of metastasis and castrate-resistant prostate cancer (CRPC) and examined if associations were modified by obesity. Finally, we examined the relationship between these outcomes and diabetes duration.

## Methods

### Study Population

After obtaining institutional review board approval, data of men who underwent RP from 1988 to 2017 at 6 Veterans Affairs Medical Centers (West Los Angeles, San Diego, and Palo Alto, CA; Augusta, GA; and Durham and Asheville, NC) were abstracted from electronic medical records into the Shared Equal Access Regional Cancer Hospital (SEARCH) cohort database (25). Men who received neoadjuvant androgen-deprivation therapy (ADT) or radiation therapy were excluded. SEARCH includes patient demographic and clinical characteristics at surgery, including surgical center, age at RP, race, height, weight, clinical stage (cT1, cT2 and cT3), D’Amico risk groups (26), cancer grade on diagnostic biopsies, preoperative and postoperative PSA, surgical specimen pathology (specimen weight, tumor volume, stage, and surgical margin status), timing of ADT, and development of metastases and PCSM.

**Figure 1.**
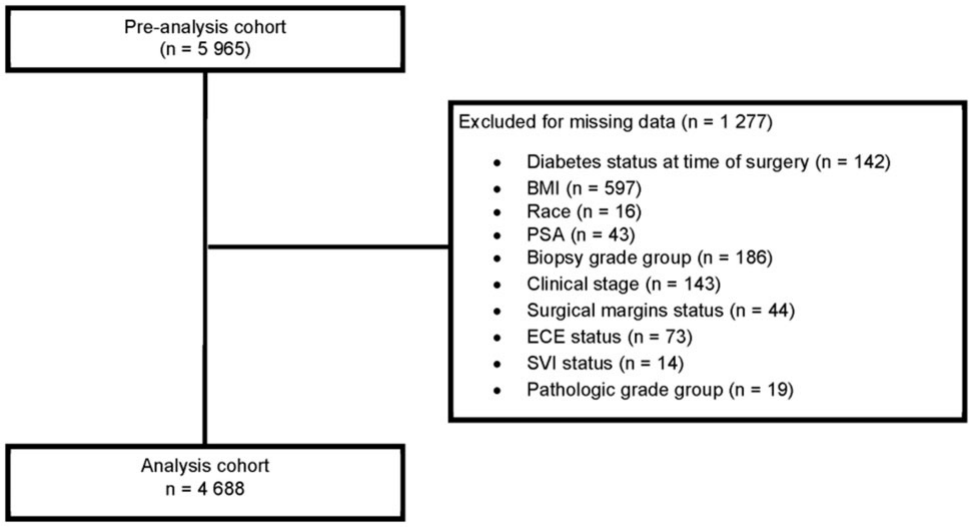
CONSORT diagram. BMI = body mass index; PSA = prostate-specific antigen; ECE = extracapsular extension; SVI = seminal vesicle invasion.

Of 5965 eligible men, we excluded men missing data at surgery, including diabetes (n = 142), body mass index (BMI: kg/m^2^; n = 597), race (n = 16), PSA (n = 43), biopsy grade group (n = 186), clinical stage (n = 143), margins status (n = 44), extracapsular extension (n = 73), seminal vesicle invasion (n = 14), and pathologic grade group (n = 19), resulting in 4688 men (see Figure 1). Excluded men were more likely to be White; be nondiabetic; have a somewhat lower BMI; have had surgery more than 10 years earlier; and have a higher median PSA, more advanced clinical stage at RP, and a longer time from RP to metastases compared with those included (Supplementary Table 1, available online).

### Assessment of Diabetes and Obesity

Diabetes status prior to RP was determined through a hand-abstracted medical chart review for HgA1c and glucose laboratory diagnosis of diabetes using the American Diabetes Association guidelines (27). If laboratory data were unavailable, documentation of self-report and prescription of antidiabetic medications were used. Duration of diabetes, defined as a continuous variable in years (year of diagnosis to RP), was calculated using laboratory data when available; otherwise, date of first antidiabetic prescription was used. If neither of these were available, chart notes were reviewed for patients’ self-reported diagnosis date. The majority (96%) of diabetic patients had type 2 diabetes.

Height and weight closest to, but within 5 years preceding RP were abstracted from the medical records for calculating BMI (kg/m^2^) and classifying men as nonobese (<30 kg/m^2^) or obese (≥30 kg/m^2^).

### Assessment of Primary and Secondary Outcomes

The primary outcome, PCSM, was defined as death with metastatic disease or progressive CRPC without other obvious or unknown causes of death. Secondary outcomes of CRPC and metastasis were defined using the PC Working Group 2 criteria: a 25% PSA increase from post-ADT PSA nadir and a PSA increase of 2 ng/mL or higher (28) and by bone scan or computer tomography imaging, respectively, performed as per attending physician’s discretion.

### Statistical Analyses

Differences in demographic and clinic-pathologic features were compared between BMI categories (obese vs nonobese) and diabetic status (diabetic vs nondiabetic) using Wilcoxon rank sum tests for continuous variables and χ^2^ tests for categorical variables.

For all outcomes, death from causes other than prostate cancer was considered a competing risk. For each outcome, we estimated the cumulative incidence function for diabetics and nondiabetics using a univariable Fine-Gray competing-risk regression model (29). Fine-Gray competing-risk regression models were also used to estimate the age-adjusted and multivariable-adjusted hazard ratios (adj-HRs) and 95% confidence intervals (CIs) for diabetes and prostate cancer outcomes (29). Multivariable models were adjusted for demographic, clinical, and pathologic factors such as age, race, log-transformed BMI, preoperative PSA (log transformed), year of surgery, surgical center, clinical stage, margins status, extracapsular extension, seminal vesicle invasion, lymph node status, and pathological grade groups (1, 2, and 3-5). The interaction term for diabetes (yes vs no) and obesity (yes vs no) was evaluated by including the cross-product term in multivariable regression models and testing for statistical significance using the Wald test. Associations between diabetes and outcomes were estimated in nonobese (BMI < 30 kg/m^2^) and obese (BMI ≥ 30 kg/m^2^) men separately in stratified analyses. For all analyses, time zero was at RP.

Among diabetic men, the associations between diabetes duration and outcomes were determined. In sensitivity analyses, outlier values for diabetes duration were identified (values > [Q3 + 1.5*IQR]), excluded, and data reanalyzed.

All tests were 2-sided with a *P* value less than*  *.05 defined as statistically significant. Statistical analyses were conducted using SAS Version 9.4 (SAS Institute, Cary, NC) and Stata 14.2 (Stata Corp, College Station, TX).

## Results

### Study Participant Characteristics

Of the 4688 men, 955 (20.4%) were diabetic and 1560 (33.3%) were obese (Table 1). Diabetic men were more likely to be Black, be older, have higher median BMI and lower PSA, have higher clinical and pathological grade and a shorter median follow-up time (all *P *<* *.001), and be in the D’Amico high-risk group (*P *=* *.002) than nondiabetic men. Obese men were more likely to be diabetic at the time of surgery and have a lower PSA, lower stage tumors (all *P *<* *.001), and higher clinical (*P *=* *.001) and pathological grade (*P *=* *.002) than nonobese men. No differences were found between diabetic and obesity status and seminal vesicle invasion, extracapsular extension, positive margins, or lymph node involvement (all *P *≥* *.06). During a median follow-up time of 7 years (estimated among surviving men), 102 men died of PC, 133 developed CRPC, and 201 had metastasis.

**Table 1.** Demographic, clinical, and pathological characteristics of patient sample stratified by diabetes and obesity status

Participant characteristics	Diabetic			Obese		
	Yes (n = 955)	No (n = 3733)	*P*	Yes (n = 1560)	No (n = 3128)	*P*
Median age (Q1, Q3), y	63 (59, 66)	62 (57, 66)	<.001^a^	62 (57.0, 65.0)	62 (58.0, 66.0)	<.001^a^
Race, No. (%)			<.001^b^			.21^b^
White	499 (52.3)	2211 (59)		874 (56)	1836 (59)	
Black	418 (43.8)	1404 (38)		633 (41)	1189 (38)	
Other	38 (4.0)	118 (3)		53 (3)	103 (3)	
Obese, No. (%)			<.001^b^			—
Yes (BMI ≥ 30 kg/m^2^)	487 (51.0)	1073 (29)		—	—	
No (BMI < 30 kg/m^2^)	468 (49.0)	2660 (71)		—	—	
BMI, median (Q1, Q3), kg/m^2^	30.2 (27.4, 33.2)	27.6 (24.9, 30.6)	<.001^a^	33.0 (31.3, 35.4)	26.3 (24.1, 28.1)	<.001^a^
Diabetic at time of surgery, No. (%)	—	—	—	487 (31)	468 (15)	<.001^b^
Duration of diabetes, median (Q1, Q3), y^c^	4 (1, 8)	0.0 (0.0, 0.0)	—	4 (1, 8)	4 (1, 8)	.72^a^
Year of surgery, median (Q1, Q3)	2009 (2004, 2013)	2008 (2002, 2012)	<.001^a^	2009 (2004, 2014)	2007 (2002, 2012)	<.001^a^
Surgery center, No. (%)			<.001^b^			<.001^b^
West LA, CA	159 (16.7)	835 (22)		260 (17)	734 (23)	
Palo Alto, CA	81 (8.5)	447 (12)		168 (11)	360 (12)	
Augusta, GA	227 (23.8)	749 (20)		361 (23)	615 (20)	
Durham, NC	217 (22.7)	731 (20)		353 (23)	595 (19)	
San Diego, CA	161 (16.9)	592 (16)		263 (17)	490 (16)	
Asheville, NC	110 (11.5)	379 (10)		155 (10)	334 (11)	
PSA, median (Q1, Q3), ng/mL	6.0 (4.6, 9.2)	6.6 (4.8, 9.9)	<.001^a^	6.1 (4.7, 9.0)	6.6 (4.8, 10.1)	<.001^a^
Clinical stage, No. (%)			.67^b^			<.001^b^
T1	593 (62.0)	2290 (61)		1017 (65)	1866 (60)	
T2 and T3	362 (38.0)	1443 (39)		543 (35)	1262 (40)	
Preoperative grade group, No. (%)			<.001^b^			.003^b^
1	347 (36.3)	1654 (44)		608 (39)	1393 (45)	
2	293 (30.7)	1057 (28)		482 (31)	868 (28)	
3	136 (14.2)	492 (13)		220 (14)	408 (13)	
4	122(12.8)	379 (10)		167 (11)	334 (11)	
5	57 (6.0)	151 (4)		83 (5)	125 (4)	
Postoperative grade group, No. (%)			<.001^b^			.01^b^
1	215 (22.5)	1067 (29)		378 (24)	904 (29)	
2	379 (39.7)	1496 (40)		664 (43)	1211 (39)	
3	199 (20.8)	673 (18)		297 (19)	575 (18)	
4	86 (9.0)	268 (7)		116 (7)	238 (8)	
5	76 (8.0)	229 (6)		105 (7)	200 (6)	
D’Amico risk group, No. (%)			.002^b^			.17^b^
Low	258 (27.0)	1224 (33)		476 (31)	1006 (32)	
Intermediate	434 (45.4)	1609 (43)		710 (46)	1333 (43)	
High	263 (27.5)	900 (23)		374 (24)	789 (25)	
Seminal vesicle invasion, No. (%)	115 (12.0)	372 (10)	.06^b^	171 (11)	316 (10)	.36^b^
Extracapsular extension, No. (%)	212 (22.2)	742 (20)	.11^b^	312 (20)	642 (21)	.67^b^
Positive surgical margins, No. (%)	393 (41.2)	1490 (40)	.49^b^	656 (42)	1227 (39)	.06^b^
Lymph node involvement, No. (%)			.27^b^			.13^b^
Yes	23 (2.4)	102 (3)		45 (3)	80 (3)	
No	568 (59.5)	2310 (62)		926 (59)	1952 (62)	
Not done	364 (38.1)	1321 (35)		589 (38)	1096 (35)	
Follow-up time, median (Q1, Q3), mo^d^	84.4 (43.9, 136.7)	94.5 (54.6, 147.9)	<.001^a^	86.4 (48.5, 140.9)	95.4 (53.9, 148.0)	<.001^a^

a Two-sided Wilcoxon rank sum test. BMI = body mass index; PSA = prostate-specific antigen; Q1 = 25th percentile; Q3 = 75th percentile; — = value not derived.

b Two-sided χ^2^ test.

c Only among patients who were diabetic at the time of surgery.

d Only among surviving men.

### Primary Outcome: PCSM and Diabetes in Obese and Nonobese Men

**Figure 2.**
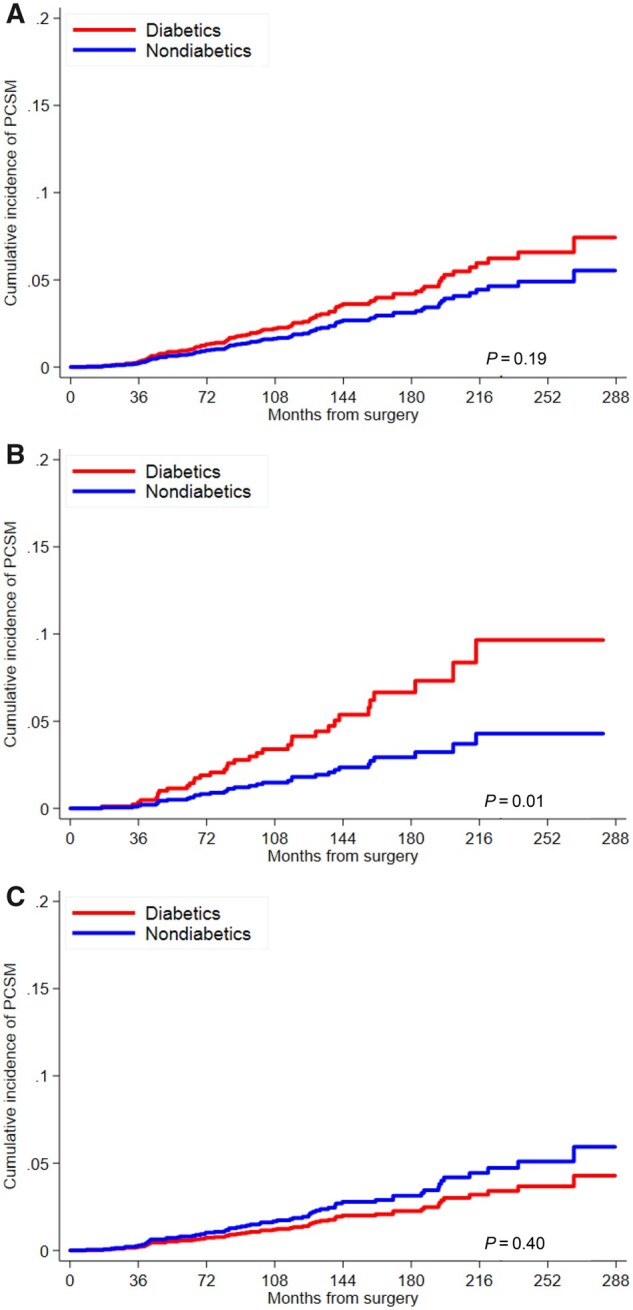
Cumulative incidence curve for risk of prostate cancer–specific mortality (PCSM) by diabetes status among **A)** all men, **B)** obese men, and **C)** nonobese men. *P* values are from univariable Fine‐Gray subdistribution hazard model and are 2-sided.

Cumulative incidence curves for PCSM risks are presented by diabetic status for all men combined and for subgroups of obese and nonobese men (Figure 2, A-C). Among all men, the risk for PCSM was increased among diabetics, but not statistically significant (adj-HR = 1.38, 95% CI = 0.86 to 2.24) (Table 2). The interaction term for obesity and diabetes in the multivariable model was statistically significant (*P*_interaction_ = .005); hence, we stratified data by obesity. Among obese men, diabetes was associated with an increased risk of PCSM (adj-HR = 3.06, 95% CI = 1.40 to 6.69), whereas among nonobese men the hazard ratio was decreased (adj-HR = 0.65, 95% CI = 0.28 to 1.49) though not statistically significant.

**Table 2.** Competing risks hazard ratios for the association between diabetes and prostate cancer–specific mortality in all men and stratified by obesity status

Diabetes status and duration	All men			Obese men			Nonobese men			*P*_interaction_ ^c^
	No. of events/No. of men	HR (95% CI)	*P*	No. of events/No. of men	HR (95% CI)	*P*	No. of events/No. of men	HR (95% CI)	*P*	
Age-adjusted										
Diabetes, No	78/3733	Referent	—	18/1073	Referent	—	60/2660	Referent	—	—
Diabetes, Yes	24/955	1.34 (0.85 to 2.12)	.21	17/487	2.25 (1.15 to 4.40)	.02	7/468	0.71 (0.32 to 1.57)	.40	.02
Duration of diabetes, y	24/955	1.04 (0.99 to 1.09)	.10	17/487	1.01 (0.94 to 1.08)	.84	7/468	1.09 (1.03 to 1.14)	<.001	.51
Duration of diabetes, y^a^	23/917	1.07 (1.00 to 1.15)	.06	17/468	1.04 (0.95 to 1.13)	.45	6/449	1.17 (1.05 to 1.29)	.003	.47
Multivariable^b^										
Diabetes, No	78/3733	Referent	—	18/1073	Referent	—	60/2660	Referent	—	—
Diabetes, Yes	24/955	1.38 (0.86 to 2.24)	.18	17/487	3.06 (1.40 to 6.69)	.005	7/468	0.65 (0.28 to 1.49)	.31	.005
Duration of diabetes, y	24/955	1.03 (0.98 to 1.09)	.24	17/487	0.99 (0.91 to 1.07)	.72	7/468	1.11 (1.02 to 1.21)	.02	.89
Duration of diabetes, y^a^	23/917	1.07 (1.00 to 1.15)	.07	17/468	1.04 (0.93 to 1.15)	.54	6/449	1.21 (0.99 to 1.47)	.06	.91

a Men with outlying values > [Q3 + (1.5*IQR)] of duration of diabetes were excluded. CI = confidence interval; HR = hazard ratio.

b Adjusted for age, race, log-transformed body mass index, preoperative prostate-specific antigen (log-transformed), year of surgery, surgical center, clinical stage, margins status, extracapsular extension, seminal vesicle invasion, lymph node status, and pathological grade group.

c Two-sided *P* value for interaction between diabetes and obesity.

Among diabetic men, duration of diabetes was not associated with PCSM in the entire cohort (adj-HR = 1.03, 95% CI = 0.98 to 1.09) or in the obese subgroup (adj-HR = 0.99, 95% CI = 0.91 to 1.07), whereas in nonobese men, longer duration was associated with an increased risk of PCSM (adj-HR = 1.11, 95% CI = 1.02 to 1.21). In sensitivity analyses, following exclusion of 38 men with outlier values (>20 years of duration of diabetes), duration of diabetes was not associated with risk of PCSM among all men or among obese men but was increased in nonobese men (adj-HR = 1.21, 95% CI = 0.99 to 1.47), although statistical significance was not attained.

### Secondary Outcomes: CRPC, Metastasis, and Diabetes in Obese and Nonobese Men

**Figure 3.**
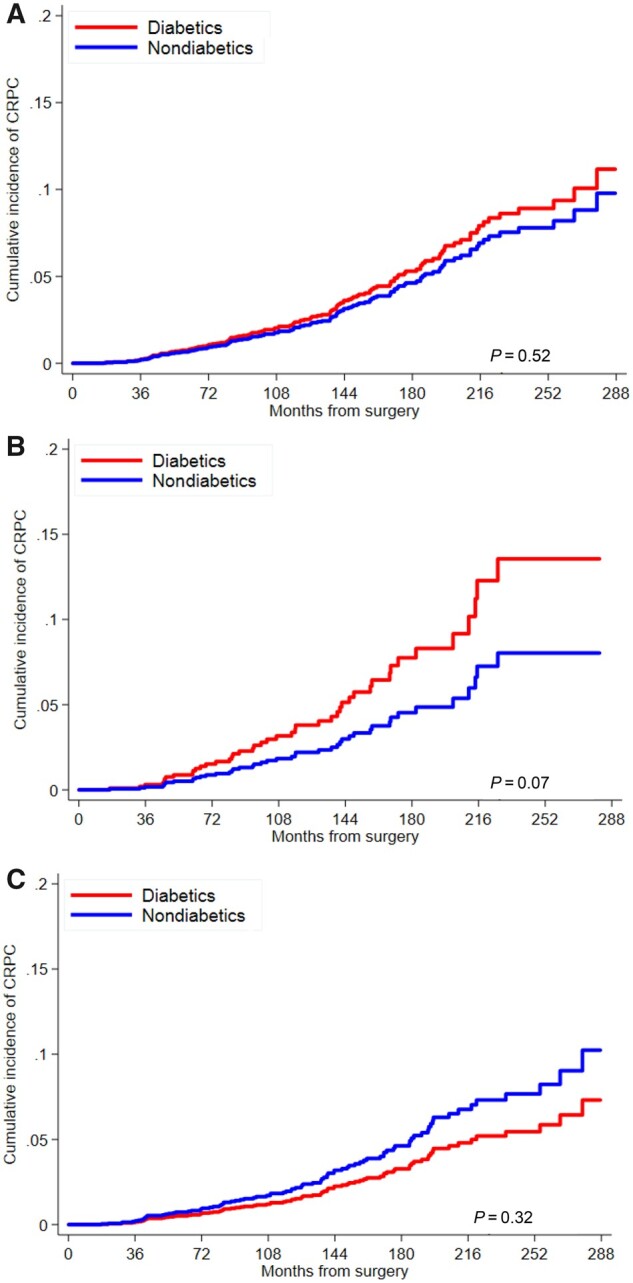
Cumulative incidence curve for risk of castrate-resistant prostate cancer (CRPC) by diabetes status among **A)** all men, **B)** obese men, and **C)** nonobese men. *P* values are from univariable Fine‐Gray subdistribution hazard model and are 2-sided.

Cumulative incidence curves for CRPC risks are presented by diabetes status for all men combined and for subgroups stratified by obesity (Figure 3, A-C). Diabetes was not associated with CRPC among all men (adj-HR = 1.05, 95% CI = 0.67 to 1.64) (Table 3); however, the interaction term for diabetes and obesity was statistically significant (*P*_interaction_ = .02). In stratified analyses, diabetes was associated with increased risk of CRPC in obese men (adj-HR = 2.14, 95% CI = 1.11 to 4.15), whereas it was decreased but not statistically significant in nonobese men (adj-HR = 0.54, 95% CI = 0.25 to 1.15). Duration of diabetes was not associated with CRPC risk in all men or in obese men but was associated with increased risk in nonobese men prior to (adj-HR = 1.09, 95% CI = 1.03 to 1.15) and following exclusion (adj-HR = 1.17, 95% CI = 1.03 to 1.31) of outliers.

**Table 3.** Competing risks hazard ratios for the association between diabetes and CRPC and METS in all men and stratified by obesity status

Diabetes status and duration	All men			Obese men			Non-obese men			
	No. of events/No. of men	HR (95% CI)	*P*	No. of events/No. of men	HR (95% CI)	*P*	No. of events/No. of men	HR (95% CI)	*P*	*P*_interaction_ ^c^
CRCP										
Age-adjusted										
Diabetes, No	106/3733	Referent	—	26/1073	Referent	—	80/2660	Referent	—	—
Diabetes, Yes	27/955	1.08 (0.71 to 1.66)	.72	18/487	1.61 (0.88 to 2.94)	0.12	9/468	0.68 (0.34 to 1.36)	.27	.06
Duration of diabetes, y	27/955	1.05 (1.00 to 1.09)	.04	18/487	1.03 (0.96 to 1.10)	0.40	9/468	1.07 (1.02 to 1.13)	.006	.47
Duration of diabetes, y^a^	25/917	1.06 (1.00 to 1.13)	.07	17/468	1.03 (0.94 to 1.12)	0.55	8/449	1.13 (1.03 to 1.22)	.006	.93
Multivariable^b^										
Diabetes, No	106/3733	Referent	—	26/1073	Referent	—	80/2660	Referent	—	—
Diabetes, Yes	27/955	1.05 (0.67 to 1.64)	.84	18/487	2.14 (1.11 to 4.15)	0.02	9/468	0.54 (0.25 to 1.15)	.11	.02
Duration of diabetes, y	27/955	1.04 (0.99 to 1.09)	.16	18/487	1.01 (0.93 to 1.10)	0.78	9/468	1.09 (1.03 to 1.15)	.002	.95
Duration of diabetes, y^a^	25/917	1.06 (0.99 to 1.13)	.12	17/468	1.03 (0.93 to 1.14)	0.62	8/449	1.17 (1.03 to 1.31)	.01	.40
METS										
Age-adjusted										
Diabetes, No	160/3733	Referent	—	38/1073	Referent	—	122/2660	Referent	—	—
Diabetes, Yes	41/955	1.08 (0.77 to 1.53)	.65	24/487	1.44 (0.86 to 2.42)	0.17	17/468	0.85 (0.51 to 1.42)	.54	.11
Duration of diabetes, y	41/955	1.05 (1.01 to 1.09)	.01	24/487	1.02 (0.96 to 1.08)	0.48	17/468	1.08 (1.04 to 1.12)	<.001	.97
Duration of diabetes, y^a^	38/917	1.05 (1.00 to 1.10)	.06	23/468	1.02 (0.95 to 1.10)	0.54	15/468	1.08 (1.02 to 1.15)	.01	.90
Multivariable^b^										
Diabetes, No	160/3733	Referent	—	38/1073	Referent	—	122/2660	Referent	—	—
Diabetes, Yes	41/955	1.01 (0.70 to 1.46)	.96	24/487	1.57 (0.88 to 2.78)	0.13	17/468	0.67 (0.38 to 1.16)	.15	.04
Duration of diabetes, y	41/955	1.04 (0.99 to 1.09)	.13	24/487	1.01 (0.94 to 1.08)	0.85	17/468	1.09 (1.04 to 1.15)	<.001	.79
Duration of diabetes, y^a^	38/917	1.04 (0.99 to 1.10)	.14	23/468	1.04 (0.95 to 1.13)	0.42	15/468	1.10 (1.01 to 1.21)	.03	.61

a Men with outlying values > [Q3 + (1.5*IQR)] of duration of diabetes were excluded. CI = confidence interval; CRPC = castrate-resistant prostate cancer; HR = hazard ratio; METS = metastasis.

b Adjusted for age, race, log-transformed body mass index, preoperative prostate-specific antigen (log-transformed), year of surgery, surgical center, clinical stage, margins status, extracapsular extension, seminal vesicle invasion, lymph node status, and pathological grade group.

c Two-sided *P* value for interaction between diabetes and obesity.

**Figure 4.**
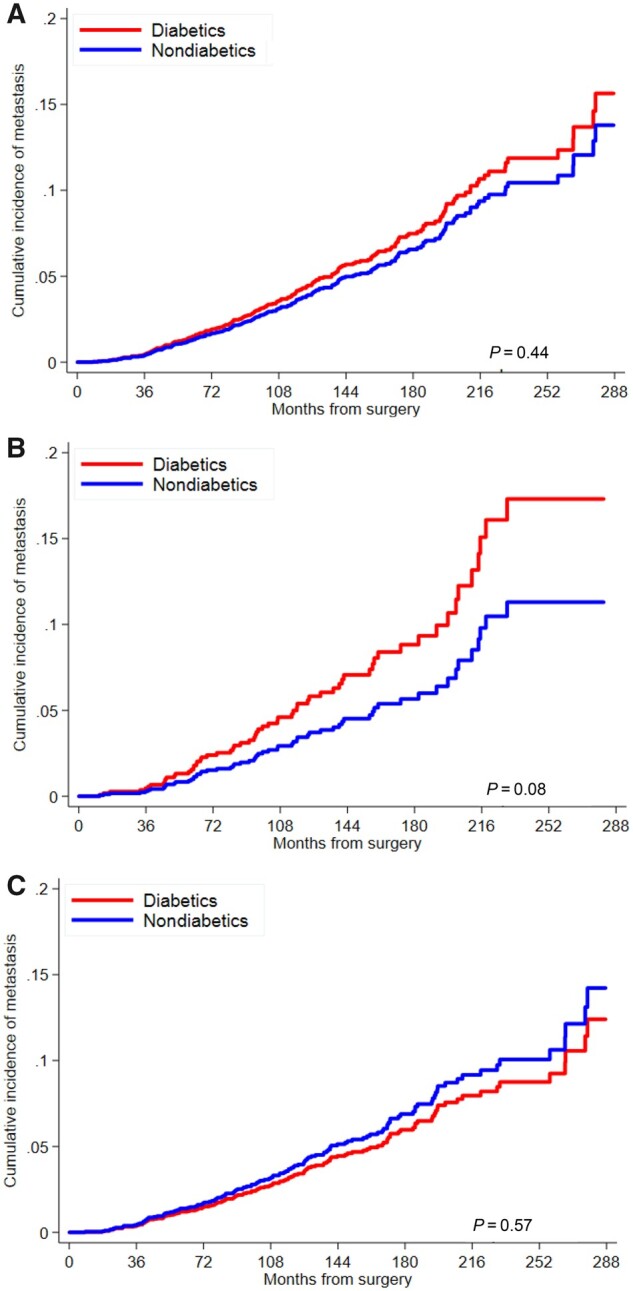
Cumulative incidence curve for risk of metastasis by diabetes status among **A)** all men, **B)** obese men, and **C)** nonobese men. *P* values are from univariable Fine‐Gray subdistribution hazard model and are 2-sided.

Cumulative incidence curves for risk of metastasis are presented by diabetic status for all men combined and for subgroups stratified by obesity (Figure 4, A-C). Among all men, diabetes was not associated with metastasis (adj-HR = 1.01, 95% CI = 0.70 to 1.46); however, the interaction term for obesity and diabetes was statistically significant (*P*_interaction_ = .04) (Table 3). In stratified analysis, although statistical significance was not attained, risk was increased in obese men (adj-HR = 1.57, 95% CI = 0.88 to 2.78) and decreased in nonobese men (adj-HR = 0.67, 95% CI = 0.38 to 1.16). Duration of diabetes was not associated with risk of metastasis in all diabetic men or in obese men; however, longer duration of diabetes was associated with metastasis in nonobese men prior to (adj-HR = 1.09, 95% CI = 1.04 to 1.15) and following exclusion (adj-HR = 1.10, 95% CI = 1.01 to 1.21) of outliers.

## Discussion

The increasing prevalence of diabetes and obesity among men in the United States highlights the pressing need to understand the interplay between these conditions and their potential roles in the rising rates of prostate cancer incidence and progression. Despite decades of research, the associations between diabetes and prostate cancer risk and outcomes remain inconclusive, although evidence for an increased risk of PCSM is emerging for preexisting diabetes (30‐32). While there is strong evidence for a link between obesity and high-grade prostate cancer risk and PCSM (17,19‐23), the association between diabetes and prostate cancer progression modified by obesity has not been well studied.

Herein, we report that among obese men, diabetes at RP was associated with an increased risk of PCSM and CRPC and suggestively associated with metastases. In contrast, in nonobese men, associations between diabetes and these outcomes were suggestive of a decreased risk. These results are consistent with our earlier findings wherein we reported that diabetes was associated with an increased risk of biochemical recurrence (24) and metastases (15) in obese, but not in nonobese, men undergoing an RP.

In subgroup analyses of diabetic men, duration of diabetes was not associated with prostate cancer outcomes in obese men, but increased risks were associated with CRPC and metastases in nonobese men. In previous analyses, we found that duration of diabetes was associated with an increased risk of metastases, however, data were not stratified by obesity (15).

We are not aware of other studies examining the modifying effect of obesity on the association between diabetes and prostate cancer progression. A study that examined the association between diabetes and mortality found no evidence of effect modification by obesity; however, mean duration of follow-up was only 4.7 years (32). As such, relevant studies pertaining to diabetes and prostate cancer risk merit consideration (8,33). In a retrospective cohort of men undergoing prostate cancer biopsies, diabetes was associated with high-grade prostate cancer in obese men (relative risk = 3.84, *P *=* *.02) but not in nonobese men (relative risk = 1.39, *P* = .46) (33). A suggestion of effect modification by obesity for diabetes and high-grade prostate cancer was also found in the Reduction by Dutasteride of Prostate Cancer Events trial (odds ratios = 1.38 and 0.35, in obese and nonobese men, respectively; *P*_interaction_ = .053), in which all men underwent protocol-driven prostate biopsies regardless of PSA levels (8). Our data are consistent with these multiple prior studies, albeit here with a PCSM endpoint, diabetes among obese men is associated with aggressive prostate cancer, whereas diabetes in nonobese men is linked with similar or lower risks of aggressive prostate cancer.

Although the relation between diabetes and prostate cancer has been described as an enigma (34), the modifying effect of obesity further complicates the pathophysiology. Diabetes is a complex disorder that is more heterogeneous than implied by the traditional dichotomous type 1 and type 2 classifications. In a Scandinavian study of newly diagnosed adult diabetics (n = 14 755), Ahlqvist et al. (35) identified 5 distinct subtypes based on cluster analysis of 6 variables (age at diabetes onset, BMI, homeostatic model assessment 2 estimates of ß-cell function and insulin resistance, glutamate decarboxylase antibodies, and glycated hemoglobin [HbA1c]). One subtype, stereotypically identified as the type 2 phenotype (comprising approximately 15% of patients in the discovery and replication cohorts), was associated with obesity and severe insulin resistance. The other 4 subtypes of diabetes included 2 severe forms characterized by insulin deficiency and low BMI and 2 diabetic phenotypes characterized by obesity and age, both associated with only mild metabolic abnormalities. This cluster-based classification has been replicated in European, Asian, and US populations demonstrating the generalizability of the subtyping to non-Scandinavian populations (36‐38). Furthermore, the subtypes have been shown to be predictive of distinctly different patterns of diabetes-related treatment response (35,36), progression (35,36) and diabetes-related complications (35‐37). Although much more needs to be understood about the clinical utility of these subtypes, the implication for prostate cancer is that the heterogeneity of diabetes, specifically the contrasting pathophysiologies of obese and severe insulin resistant vs low BMI and insulin deficient phenotypes, may explain the obesity-related differences in associations between diabetes and prostate cancer outcomes.

Obese diabetic men in our study may represent the subtype with severe insulin resistance at greater risk of diabetes-related complications (35). Insulin resistance promotes increased levels of circulating endogenous insulin and insulin-like growth factor levels (21) associated with increased risks of high-grade prostate cancer (39) and PCSM (20). Aggressive prostatic tumors have been shown to have an increased number of insulin receptors that activate a cascade of signal transduction pathways, creating a favorable environment for tumor growth and metastases (21,39‐41). Nonetheless, the obese diabetic subgroup in our study may also include mild obesity and age-related diabetes subtypes with only moderate levels of insulin resistance and metabolic abnormalities (35,37), which would be predicted to be less strongly linked with PCSM. As such, our results may underestimate the potential for severe insulin-resistant diabetes to increase prostate cancer progression.

Among nonobese men in our study, 15% were diabetic. This diabetic subgroup may represent the low BMI, insulin-deficient subtype described by Ahlqvist et al. (also approximately 15%) (35), who may be less likely to have elevated circulating insulin and insulin-like growth factor levels. However, they may also be more likely to have poor glycemic control with relatively shorter time to requiring insulin (35,37). We previously showed that poor glycemic control as measured by HbA1c was associated with increased risks of metastases and CRPC in diabetic men (16). In addition, exogenous insulin has been associated with various adverse outcomes, including overall cancer mortality (42,43). The results in nonobese men, suggestive of an inverse association between diabetes and prostate cancer progression and increasing risk with duration of diabetes, may reflect an interplay of these factors.

Although the underlying pathophysiology described above is plausible, we cannot rule out the possibility of bias playing a role in our findings. First, poor prostate cancer prognosis in obese men has been proposed to result from low PSA values owing to hemodilution potentially delaying diagnosis (20,44,45). In men with poorly controlled diabetes, PSA can also be lower because of the PSA-lowering effect of glucose (7). Second, antidiabetic medications have been reported to lower PSA levels in diabetic men (10), potentially decreasing the rate of referral for biopsy, delaying diagnosis and treatment, and ultimately leading to more aggressive disease. However, a study among prostate cancer-free men failed to find serum PSA lowering with metformin, sulfonylurea, or insulin use (11). Nonetheless, in our analyses, we adjusted for PSA levels, clinical stage, and pathological grade group, thus our results are suggestive of underlying biological differences rather than the biases described above.

Limitations of this study warrant mention, notably the modest number of prostate cancer events, particularly among nonobese diabetic men. In future studies, the association between diabetes and prostate cancer outcomes should be studied across multiple categories of BMI in larger studies rather than across 2 categories of BMI only. In addition, only weights closest in time prior to RPs were ascertained. Future studies are needed to assess how long-term BMI changes impact prostate cancer progression. We were also unable to adjust for diet and physical activity; however, the evidence for their impact on prostate cancer progression independent of obesity is currently weak (19). Furthermore, we did not examine the severity of diabetes or the use of antidiabetic medications. In addition, as no quality-of-life data were collected, we are unable to comment on the impact of diabetes or obesity on these outcomes. Finally, we caution that our results may not be generalizable to men undergoing other prostate cancer treatment modalities. Although associations between diabetes and outcomes have been studied in men undergoing radiation, such studies have not been stratified by BMI and, hence, merit investigation in future research. Additionally, large prospective studies with detailed antidiabetic medication prescription, glycemic, metabolic, and molecular biomarker data are needed to better understand the nature of heterogeneity in diabetes, its interaction with obesity, and its link to prostate cancer progression and mortality.

Importantly, our study has several noteworthy strengths. Although SEARCH is a retrospective cohort, highly accurate clinical data, documented as prospectively occurring events, are manually abstracted from medical records ensuring the correct temporal order of exposures (diabetes and obesity status) in relation to outcomes. Additionally, the Veterans Affairs health-care system ensures uniform equal access to medical coverage of all its members.

To conclude, in our study of men undergoing RP, diabetes was associated with an increased risk of PCSM and progression in obese but not in nonobese men. Our results highlight the need to aggressively curtail and reverse the increasing prevalence of obesity to avert a potential increase in the morbidity and mortality of prostate cancer survivors known to be at risk of secondary malignancies (46) and cardiovascular disease (47).

## Funding

This work was supported the National Cancer Institute at the National Institutes of Health (Grant Number R01CA231219 to WJA).

## Notes

**Role of the funder:** The National Institutes of Health had no role in the design, conduct, analysis, or reporting of the study results and did not participate in the design and conduct of the study, collection, management, analysis, and interpretation of data, preparation, review, or approval of the manuscript, or the decision to submit the manuscript for publication.

**Disclosures:** The authors have no disclosures or conflicts of interest to report related to this work.

**Author contributions**: SJF, IC, TO, AE, and LH conceptualized and designed the analysis; IC provided supervision; SK and IC wrote the original draft of the manuscript; TO, SJF, and IC contributed to the data analysis and/or interpretation; SJF and WJA obtained funding; SJF, WJA, CJK, CLA, MRC, ZK, MKT, and AE contributed to data collection methods; all co-authors contributed to critical review and approved of the version.

**Prior presentation:** Abstract presentation at the American Urological Association Annual Meeting held virtually, May 16, 2020 due to COVID.

## Data Availability

The data and software code upon which the conclusions of this article rely will be made available upon reasonable request to the corresponding author.

## Supplementary Material

pkab023_Supplementary_Data
