# Supplementary material for: Diabetes and Prostate Cancer Outcomes in Obese and Nonobese Men After Radical Prostatectomy
Source: JNCI Cancer Spectr. 2021 Mar 9;5(3):pkab023. doi: 10.1093/jncics/pkab023 (PMC8220304; doi:10.1093/jncics/pkab023)
Supplement: pkab023_Supplementary_Data [file pkab023_supplementary_data.pdf]

Supplementary Table 1. Comparison between excluded and included patients.

	Excluded		
	Yes (N=1277)	No (N=4688)	p value
Age, *median (Q1, Q3)*	65 (61, 69)	62 (58, 66)	<0.001[a]
Race, *n (%)*			<0.001[b]
White	1007 (80)	2710 (58)	
Black	214 (17)	1822 (39)	
Other	35 (3)	156 (3)	
Missing	21	0	
BMI (kg/m^2), *median (Q1, Q3)*	27.3 (24.5, 30.4)	28.1 (25.4, 31.3)	<0.001[1a]
BMI (kg/m^2), *n (%)*			0.007[b]
<30	408 (72)	3128 (67)	
>=30	156 (28)	1560 (33)	
Missing	713	0	
Diabetic at time of surgery, *n (%)*			<0.001[b]
Yes	139 (12)	955 (20)	
No	996 (88)	3733 (80)	
Missing	142	0	
Year of surgery, *median (Q1, Q3)*	1994 (1992, 1997)	2008 (2003, 2012)	<0.001[a]
Surgery center, *n (%)*			<0.001[b]
West LA	83 (6)	994 (21)	
Palo Alto	103 (8)	528 (11)	
Augusta	81 (6)	976 (21)	
Durham	158 (12)	948 (20)	
San Diego	512 (40)	753 (16)	
Asheville	340 (27)	489 (10)	
PSA (ng/mL) , *median (Q1, Q3)*	7.1 (4.6, 12.0)	6.4 (4.8, 9.7)	0.001[a]
Clinical stage, *n (%)*			<0.001[b]
T1	339 (27)	2883 (61)	
T2/T3	938 (73)	1805 (39)	
Pre-op grade group, *n (%)*			<0.001[b]
1	523 (65)	2001 (43)	
2	150 (19)	1350 (29)	
3-5	132 (16)	1337 (29)	
Missing	472	0	
Seminal vesicle invasion, *n (%)*			0.005[b]
Yes	157 (13)	487 (10)	
No	1026 (87)	4201 (90)	
Missing	94	0	

	Excluded		
	Yes (N=1277)	No (N=4688)	p value
Extracapsular extension, *n (%)*			0.005[b]
Yes	268 (24)	954 (20)	
No	841 (76)	3734 (80)	
Missing	168	0	
Positive surgical margins, *n (%)*			0.100[b]
Yes	445 (38)	1883 (40)	
No	740 (62)	2805 (60)	
Missing	92	0	
Lymph node involvement, *n (%)*			0.248[b]
Yes	24 (2)	125 (3)	
No	781 (61)	2878 (61)	
Not done	472 (37)	1685 (36)	
Post-op grade group, *n (%)*			<0.001[b]
1	427 (47)	1282 (27)	
2	270 (30)	1876 (40)	
3-5	209 (23)	1530 (33)	
Missing	371	0	
Months from surgery to metastasis, *median (Q1, Q3)*	141.1 (66.6, 213.4)	92.5 (50.8, 145.2)	<0.001[a]

[a]Wilcoxon rank sum test; [b]Chi-Square test.
Missing data were not included in computing statistical tests.
